# Circular Economy Business Models: a Repertoire of Theoretical Relationships and a Research Agenda

**DOI:** 10.1007/s43615-021-00133-x

**Published:** 2021-11-24

**Authors:** Roberta De Angelis

**Affiliations:** Cardiff Business School, Aberconway Building, Colum Drive, Cardiff, Wales CF10 3EU UK

**Keywords:** Circular economy, Business model, Theory, Systems thinking, Competitive advantage, Transition

## Abstract

The shift towards a more resource efficient circular economy has become a necessity in the wake of current ecological, economic and social sustainability challenges. Mirroring circular-related developments in policy and business quarters, the circular economy literature is growing as a distinct field of academic enquiry. Yet, the conceptual and theoretical foundations of circular economy thinking need consolidation. Drawing from strategic management, sustainability transitions and systems theories, this article establishes some theoretical anchoring for circular economy business models. It finds that circular business models contribute to an understanding of both competitive advantage and the systemic nature of business. It also develops a future agenda for management research at the interface between the circular economy and business models.

## Introduction

The state of the planet Earth is appalling. Biodiversity is falling, with 1 million species at risk of extinction; deserts are spreading; forests are being lost; coral reefs are dying; carbon emissions keep rising, and oceans are devastated by overfishing and plastic waste [1]. *“We need to learn how to work with nature rather than against it”*, counsels Sir David Attenborough – a British broadcaster and naturalist – in his latest documentary *A Life on Our Planet* [2]. Given the urgency of the ecological crisis, what can steer society towards a more harmonious relationship with nature, one wherein all living systems can flourish and prosper?

Since the early writings of Boulding (1966) [3] in *The Economics of the Coming Spaceship Earth,* a cyclical rather than a linear pattern of materials use has become even more urgent and powerfully championed by the concept of the circular economy recently. Known as an economy that is “restorative and regenerative by intention and design” [4, p. 7] seeking to eliminate the concept of waste and pollution, maintain products and materials in use and regenerate natural systems [5], the circular economy (CE) has emerged as a convincing win–win scenario for an economy thriving within the humanity’s safe-operating space represented by planetary boundaries [6]. Espousing nature material efficiency principles, the CE has been embraced across different quarters to address current environmental, economic, and social sustainability challenges, including climate change, depletion of finite natural reservoirs, and a broken economy in the aftermath of the COVID-19 pandemic [7, 8].

Mirroring developments in business and policy contexts, CE thinking has entered the scholarly literature contributing to the emergence of a distinct field of academic enquiry, particularly from 2015 onwards [9]. Concepts and theories evolve through three different stages: introduction and elaboration, evaluation and augmentation, and consolidation and accommodation [10]. Currently, the CE field sits within stage one. Understanding of CE is multifaceted, lending itself to some conceptual ambiguities and, so, research that consolidates knowledge, identifies and builds a common theoretical background, is welcome [11–13]. When concepts are not defined clearly, effective communication, theory building and creativity are in an impasse, and this compromises the development of any field [14, 15]. Concurring with Bansal and Song (2017) [16], here it is argued that “an academic field’s development is aided by a consensual research” (p. 106).

To contribute towards conceptual and theoretical clarity in the CE field, this article establishes some theoretical anchoring for advancing the study of circular business models (CBMs). In line with management scholars’ rising interest in environmental sustainability research [17, 18], CBMs have increasingly become the subject of academic enquiry [19]. This is not surprising considering that CE thinking challenges substantially the linear logic of value creation, and so it becomes crucial to investigate innovative ways of doing business to create and capture value in a CE. Hence, this article asks: how can the theoretical coupling of CBMs be advanced?

To answer this question, this research draws on the business model (BM) literature because CBMs are among the offsprings of the BM concept. Therefore, it is pertinent to link the study of the theoretical foundations of CBMs to the theoretical underpinnings of the mainstream BM literature. Working as “the webbing between theories” [20, p. 7] including the resource-based-view of the firm [21], the demand-side perspective [22] and the dynamic capability view [23], BMs and BM research are currently positioned within the strategic management literature [20]. Hence, the repertoire of theories used in this article includes strategic management lenses, and, particularly, the natural-resource-based view of the firm [24]. Nonetheless, it also extends beyond those lenses and comprises sustainability transitions and system theories, which are not new to corporate sustainability and CE studies [25–28].

The remainder of this article is organised in the following sections. Next, the CE and the CBM concepts are briefly illustrated. The subsequent sections discuss the basis for establishing the theoretical coupling of CBMs and explain the rationale for employing strategic management theories as well as transitions and systems theories to advance the theoretical anchoring of CBMs. Finally, the research contributions, implications for theory and practice as well as future lines of enquiry are illustrated.

## Circular Economy and Circular Business Models

*“We cannot solve our problems with the same thinking we used when creating them*,” Albert Einstein famously argued. The negative environmental externalities associated with wasteful production and consumption systems cannot be solved by relying on the same linear thinking that has caused them. What else can instead?

Supported by a sound economic rationale, CE thinking has emerged as a promising and viable vision to move towards a more resource-efficient and resilient economy. It is estimated that the CE could offer a $4.5 trillion opportunity by 2030 and several businesses are starting to capture their share via reduced costs, better customers’ and employees’ relationships, enhanced sales and mitigation of risks associate with linear-operating BMs [29]. Furthermore, applying CE principles in just five key areas (cement, aluminium, steel, plastics, and food) can eliminate 9.3 billion tonnes of CO_2_ emissions in 2050 – which equals to cutting current emissions from all transport to zero [30]. Unsurprisingly, the CE concept has been embraced by several leading corporations and small innovators as well as by local, national and supranational governments. A number of CE initiatives involving different stakeholders on different scales are emerging. One of these is the New Plastics Economy – a global initiative seeking to build a CE for plastics led by the Ellen MacArthur Foundation. Within this, companies such as PepsiCo, Unilever, and The Coca-Cola Company have committed to using 100% reusable, recyclable, or compostable packaging by 2025 [31]. The European Commission’s Green Deal – the 2020’s new agenda for a sustainable European economy – includes the Circular Economy Action Plan, which aims to accelerate the transition towards the CE with policies empowering consumers and governing waste generation and sustainable products, with a focus on high-impact sectors as textiles, construction and electronics [32].

For their potential to create multiple forms of value, CBMs have also become the subject of business innovation and academic research [33, 34]. Scholarly literature has explored categorisation tools [35, 36], practical implementation [37–39], benefits, drivers and barriers to implementation [40–42] and conceptualisations [43]. However, with few exceptions [e.g. 26, 44–46], theoretically anchored CBMs studies are limited. In fact, Geissdoerfer et al. (2020) [43] lament that “despite the importance of the circular business model notion, there is considerable lack of clarity about its theoretical conceptualisation” (p. 1). The status of the theoretical traits of CBMs is similar to that of mainstream BMs with Prescott and Filatotchev (2021) [47] highlighting that “the popularity of the business model phenomenon has outpaced its theoretical development” (p. 517).

Hence, it becomes pertinent to ask: how can the theoretical coupling of CBMs be advanced? The remainder of this article is devoted to providing an answer to this question.

## Theoretical Coupling of Circular Business Models

Bansal and Song (2017) [16] argue that “no social construct is completely de novo. Therefore, for construct clarity, researchers must discriminate a focal concept from similar existing constructs by drawing theoretical linkages with relational constructs, which forms a conceptual network within disciplinary fields” (p. 111). CBMs are among the offsprings of the BM concept. Hence, following Bansal and Song’s (2017) [16] line of argument and for the purpose of construct clarity, theoretical linkages between the two concepts can be established. Such a coupling is welcome to cross-develop both constructs [48].

Foss and Saebi (2018) [49] argue that one dimension of construct clarity is to determine exactly the explanatory role of BMs, i.e. do they contribute to an understanding of competitive advantage? Do they highlight the systemic nature of businesses? For theory building, “a construct should reflect the explanatory purposes to which the construct is put to use” (p. 14). Similarly, Ritter and Lettl (2018) [20] counsel that “it is important for every research field to define its domain as well as its connections to other theories to determine the areas that the theory covers” (p. 4). Accordingly, they position BM research within the strategic management literature since the BM concept has the potential to enrich the strategic management field. This is the case because it acts as “the webbing between theories” (p. 7) including the resource-based view of the firm [21], the demand-side perspective [22] and the dynamic capability view [23].

Strategy research has mostly emphasised how firms capture value [50]. The BM concept, instead, with its simultaneous focus on value creation (demand side) and capture [51, 52], extends the mainstream understanding of how firms create and capture value [53], according to which value is created through a focus on the supply side of transactions, i.e. either resources [21] or value chain activities [54]. Therefore, BM literature suggests that firms must create value for their customers and have mechanisms in place to capture parts of that value [52]. The all-encompassing nature of the BM concept is exemplified by Teece (2010) [55] with his definition of the BM as the “architecture of value creation, delivery, and capture mechanism” (p. 172).

In line with the BM literature, conceptualisations of the CBM focus on value creation, delivery, and capture. Accordingly, a CBM is defined as “one in which a focal company, together with partners, uses innovation to create, capture, and deliver value to improve resource efficiency by extending the lifespan of products and parts, thereby realizing environmental, social, and economic benefits” [56, p. 6]. Therefore, for the purpose of this research, it is appropriate to establish a construct relationship with theories in the strategic management field.

Nonetheless, this article theoretical coupling of CBMs extends beyond the strategic management field. Stead and Stead (2019) [57] question the suitability of conventional strategic management frameworks and models at portraying a comprehensive picture of the current business reality, wherein value creation and capture cannot prescind from considering the embeddedness of business within socio and natural ecosystems. Similarly, Post et al. (2020) [58] argue that leading management theories may not be fully appropriate to analyse the current business context due to grand societal challenges affecting the management problems under investigation. The mismatch between the theoretical lenses in use and the context investigated can further exacerbate the long-debated issue of the relevance of management research [58, 59]. As a result, this article repertoire of theoretical relationships comprises sustainability transitions and systems theories, which have already been applied in corporate sustainability and CE studies [25–28].

Next, the theoretical coupling of CBMs is developed. Following Foss and Saebi’s (2018) [49] approach in BM research, the line of argument is organised around the explanatory purpose of CBMs: do CBMs contribute to an understanding of competitive advantage? And also, do CBMs contribute to an understanding of the systemic nature of business?

## Circular Business Models and Competitive Advantage

To explain why CBMs can be a source of competitive advantage, this article draws on the resource-based view (RBV) of the firm [21] and one of its most prominent spin-offs, i.e. the natural-resource-based view (NRBV) of the firm [24]. The rationale for this choice is justified on the premise that the RBV of the firm is one of the most influential theories in the strategic management field, and the NRBV of the firm has been applied extensively in corporate sustainability literature [60].

In seeking to explain how competitive advantage can be attained and sustained in the long term, the RBV of the firm focuses on a company’s internal environment. Resources that are rare, inimitable, non-substitutable and a source of value, lead to the attainment of a sustained competitive advantage [21]. From an environmental sustainability angle, the RBV of the firm attracted some criticism. Claiming that a firm’s response to the changing ecological circumstances is relevant to building and sustaining its competitive advantage in a natural-resource-constrained world, Hart (1995) [24] proposed a natural-resource-based approach. He argued that in responding to environmental challenges, companies develop new capabilities with each of these having different drivers and outcomes in terms of competitive advantage: *pollution prevention*, *product stewardship *and *sustainable development*. *Pollution prevention* is the least ambitious strategy aiming at preventing negative environmental externalities (e.g. emissions, waste). It can be a source of competitive advantage because it reduces, for instance, inputs and legal compliance costs [24]. *Product stewardship* has a more comprehensive focus as it concentrates on the entire activity system of a company. Competitive advantage results from gaining exclusive access to resources and setting new standards (ibid.). *Sustainable development* has the broadest scope: rethinking production processes so that environmental harm is not simply reduced but avoided as well as addressing social sustainability concerns. Competitive advantage can result from accessing new markets, i.e. the bottom of the pyramid, with products targeted to people living in the developing world (ibid.).

## Resource-based Theories and Circular Business Models

The literature on CE has established some linkages between CE principles/CBMs and competitive advantage. Lacy and Rutqvist (2015) [61] term “circular competitive advantage” the competitive advantage that companies can achieve by including CE principles in the design of innovative BMs. Similarly, Prieto-Sandoval et al. (2019) [62] suggest that by providing customers with greener products and services, CE principles can be instrumental to the achievement of competitive advantage in the form of enhanced reputation and profitability. Tonelli and Cristoni (2019) [63] add that the shift towards a CE leads to the development of new core competencies along the value chain and better organisational performances that reduce costs, improve efficiency, and respond to increasingly demanding regulatory pressures and customers’ expectations. Analogously, Moric et al. (2020) [64] and Hofmann and Jaeger-Erben (2020) [65] highlight that CE-informed business strategies enhance resource productivity, lower the demand for energy and raw materials inputs and mitigate resource price volatility, thereby improving competitiveness and profitability. Furthermore, an explicit link between resource-based theories and CBMs has been put forward by some scholars [e.g. 44, 62, 66]. In the light of the escalating ecological crisis and following Hart’s (1995) [24] line of argument, De Angelis (2018) [44] notes that the attainment of a sustained and sustainable competitive advantage requires the development of resources and capabilities for managing natural resources more efficiently and effectively, which is at play when implementing BMs based on CE principles. Hence, she argues that competitiveness logics support the case for adopting CBMs.

While resource-based theories help with the positioning of the CBM concept within the strategic management field linking CBMs to competitive advantage, they are only partially fit for the purpose of the theoretical coupling of CBMs. The NRBV of the firm rightly portrays firms in relation to a wider organisational context [67]. Yet, it also seems to neglect the quest for a deeper recognition of the natural environment. Bansal and Knox-Hayes (2013) [68] warn that “framing the natural environment as merely a context or issue, rather than focusing on its unique attributes, may actually prevent researchers from making the frame-breaking insights needed to reconcile the needs of the business with the demands of the natural environment” (p. 62). Similarly, Starik and Kanashiro (2013) [69] criticise the NRBV of the firm for its reductionist view of the natural environment arguing that “nature is not only a collection of disaggregated resources for human business use” (p. 15). Analogously, Williams et al. (2017) [28] argue that resource-based approaches fall short of addressing the interconnectedness existing between the firm and its socio-ecological context, which, by contrast, is found in systems thinking.

Therefore, to uncover the theoretical relations of CBMs, what can be learnt from other scholarly fields? Recalling Foss and Saebi’s (2018) [49] approach in BM research, and so the issue of the explanatory purpose of CBMs, in the next sections this article asks: do CBMs contribute to an understanding of the systemic nature of business? The systemic aspect is explored under two interpretive frames: a “worldview” frame in the sense that under a circular logic, businesses are part of the wider system within which they operate, and a coevolutionary frame since the implementation of CBMs requires multiple forms of innovations that span organisational boundaries.

## Circular Business Models and the Systemic Nature of Business

To analyse whether CBMs contribute to understand the systemic nature of business, the analysis draws on sustainability transitions and systems theories, respectively. This is the case because they have already received some applications in corporate sustainability and CE studies [e.g. 25-28].

## Circular Business Models Under Coevolutionary Lenses

The entrepreneurial initiative of business leaders and innovative BMs are certainly essential for the emergence of a CE. The centrality of the BM concept to bring about the transition towards the CE is clearly evidenced in the CE literature [e.g. 70–72]. Webster (2013) [27] argues that CE is “led by business for a profit within the rules of the game” (p. 543). Hofmann and Jaeger-Erben (2020) [65] view CBMs as Schumpeterian instruments “for an ecological-oriented process of creative destruction” (p. 2772).

However, the attainment of such a complex system change involves more than just business ingenuity and initiative. CBMs entail a focal company to collaborate with its ecosystem partners to create, deliver and capture value [33], since value creation in a CE is “an inherently boundary spanning activity – requiring cross functional teams and new or enhanced forms of external collaboration and system configurations” [73, p. 2]. Yet, while it is recognised that moving towards the CE is a complex endeavour requiring multiple innovations across different levels, little is said in terms of how such a transition might unfold [74]. The move towards the CE can be configured as a socio-technical transition, i.e. “a combination of technical, organizational, economic, institutional, social–cultural and political changes” [75, p. 2]. These transitions are complex, develop over the long-term and involve many actors [76].

Given the multilevel and multidimensional features of sustainability issues, it is not surprising to see that transitions theories have emerged within the context of sustainability research [see 77 for a review]. Sustainability transitions research is guided by the principle that grand societal challenges such as depletion of natural resources and climate change cannot be solved by means of incremental innovation and technological solutions only, but they require radical changes in production and consumption patterns across heat, electricity, buildings, mobility and agro-food socio-technical systems [77]. Theoretical frameworks used in the sustainability transitions studies include the Multi-Level perspective, the Technological Innovation System approach, Strategic Niche Management and Transition Management [78]. They are based upon a set of shared constructs such as *socio-technical systems*, *niches* and *regimes*, although they study sustainability transitions in different ways and for different purposes [25]. Socio-technical *regimes *refer to “entrenched shared rules and institutions” [79, p. 189] and *niches* to “a protected space where experimentation with radical innovations can eventually bring about changes to sociotechnical regimes” [25, p. 17].

A core theme within transitions research is the relationship between stability and change [77]. Sources of inertia are existing structures that foster stability, lock-in and path dependence and that perform as barriers to change [25]. Socio-technical regimes are one source of inertia; sources of change, instead, are activities, forces and elements that foster transformation through technological and organisational innovations (ibid.). Landscape pressures and niche-level activities (e.g. experimentation with radical innovation) combine acting as sources of change [80], and this combination can result in radical transformations, or “transition pathways” eventually [81].

The business wider system interconnectedness underlying CBM implementation involving many actors beyond the boundaries of the single organisation, reveals the suitability of the CBM concept to highlight the systemic nature of business and the appropriateness of transitions lenses to develop the theoretical anchoring of CBMs. In fact, recent research confirms the opportunity of this coupling. CBM innovation is viewed as a dynamic process involving collaboration across systems of interdependent actors: “no single actor can drive institutional change and innovate business models in isolation and the systemic alignment processes that shape business models can only be understood when viewed from various system levels (e.g., micro, meso, and macro levels of aggregation)” [82, p. 614]. At the same time, “there are five interconnected sub-systems that need to be considered for supporting transitions to CE, namely, resource flows and provisioning service; governance, regulatory framework and political landscape; business activities and the market; infrastructure and innovation; and user practices” [83, p. 1].

## Circular Business Models Under Systems Theory

According to Meadows (2009) [84], a system is “an interconnected set of elements that is coherently organized in a way that achieves something” (p.11). Systems theory challenged the mechanistic, Cartesian’s worldview underlining all science fields until the late twentieth century, according to which from the properties of the parts it is possible to understand the behaviour of the whole [85, 86]. By contrast, in systems theory, there is a shift in emphasis from the parts to the whole, meaning that the properties of the whole cannot be reduced to those of the parts. Within systems, elements are interconnected in a way that they produce their own behaviour over time, and there are constant feedback loops and flows of information across the elements in the system [84]. Two major schools of thought are associated with systems theory: general systems theory and cybernetics, whose founding fathers are Ludwig von Bertalanffy and Norbert Wiener, respectively [87]. This holistic perspective brought by systems theory has been termed as ‘systemic’ and the thinking it implies as ‘systems thinking’ [86].

What are the main implications of systems theory from the business-natural perspective? From a business-natural environment interface, organisations cannot ignore the biophysical reality upon which they depend for their survival. The 1995’s special topic forum on ecologically sustainable organisations in *The Academy of Management Review* [e.g. 88–90] encouraged management scholars to espouse systems thinking by reconceptualising organisations as embedded within the broader socio-ecological system. To what extent does CE thinking encourage viewing organisations as part of a wider system?

The CE promotes the reintegration of the economy within ecology; it is restorative and regenerative from the outset, and it seeks to decouple economic growth from environmental depletion, hence, providing multiple forms of value [4, 91]. Furthermore, in a CE stocks and flows of resources (money, materials, information, and energy) interact with each other, and so feedback loops are acknowledged [92]. It is also the case that in a CE products are designed considering their interaction with economy and ecology along their entire useful life (ibid.).

CE thinking draws significantly from systems thinking. It encourages viewing businesses as part of the wider system within which they operate, and this relationship is acknowledged at all levels. Likewise, CBMs, shift the firm centric view of value creation and capture to the systemic level, providing a much more holistic value creation mechanism, which spans the economic sphere to include the social and environmental spheres [56, 82]. Therefore, the systemic thinking underlying CE principles and practices points to the appropriateness of system theory to develop the theoretical anchoring of CBMs and highlights that the CBM concept is useful to portray the systemic nature of business.

## Discussion

This research unveils that the theoretical coupling of CBMs can be informed by an interpretive repertoire comprising strategic management theories as well as transitions and systems theories.

The strategic management perspective is useful to highlight the competitiveness logics underlying CBMs and it is pertinent to advance the current understanding of CE business strategies. Research on the micro level and so on the organisational dimension of the CE is limited [93, 94]. Accordingly, Hofmann and Jaeger-Erben (2020) [65] lament that “the organizational dimension of CBM innovation remains uncharted territory” (p. 2771) and Eikelenboom and de Jong (2021) [95] that “little is known about the organizational attributes that can assist businesses in integrating circularity in their strategies” (p. 1). Future research questions for advancing the study of CBMs from a strategic management perspective could include: which managerial and organisational resources and capabilities explain the emergence and implementation of CBMs? Are these resources and capabilities valuable, rare, inimitable, and non-substitutable and, thereby, a source of sustained competitive advantage? How can the paradoxical demand between securing a competitive advantage at the firm level and collaboration across the value chain be solved?

Turning to transitions theories, these are very pertinent to highlight the complexity of the systemic innovations that are needed to support the emergence and scaling up of innovative CBMs. Only recently, transitions lenses have been applied in CE research [96]. Cramer (2020) [97] uses transition management theory to explain the implementation of the CE programme in the Amsterdam Metropolitan Area. Kern et al. (2020) [98] use the Deep Transitions framework to explore why and how the EU embraced the CE concept and promoted its diffusion on a global scale. Yet, further application of transitions theories in CE research and, particularly, from a BM perspective, is encouraged by Bocken et al. (2021) [96], who argue that CBM experimentation could benefit from integration with research in transitions studies. Coupling the organisational perspective, from a BM angle, with transitions lenses, is, therefore, useful to illustrate how the interplay between agency and structure results in a CE transition. Hence, CBMs can be used to advance sustainability transitions studies. In fact, while BMs can be seen as both a source of stability and change [25, 77], businesses and BMs have not been the subject of extensive research in the transitions literature [99, 100] with Ruggiero et al. (2021) [101] highlighting that “BMI behaviours and strategies in the context of multi-niche and multi-regime interactions are largely missing” (p. 2).

Additionally, transitions literature can inform the development of the theoretical coupling of CBMs. In fact, although innovation in BMs for a CE is the result of individual/organisational agency, the structure must be aligned so that CBMs can effectively emerge and reach the desired scale. Future research questions for advancing the study of CBMs from a transition theories perspective could include: under what circumstances CBMs break inertia and bring about change in established socio-technical regimes? What is the mediating role of regimes and landscapes in the emergence and implementation of CBMs?

Finally, as the CE draws on systems thinking, systems theory is a pertinent perspective to further advance the theoretical coupling of CBM studies and better inform the relationship between CE and CBMs, which, according to Rovanto and Bask (2020) [38] “is still rather informal and ill-defined” (p. 5). Future research questions for advancing the study of CBMs from a systems theory perspective could include: how exactly do CBMs contribute to maintaining stocks of natural resources? Under what circumstances does systems thinking flourish and inspire the process of multiple value creation? How can systems thinking be shared across the value chain? What mechanisms should be in place to measure the value captured in non-economic terms?

## Theoretical and Practical Contributions

The development of any research field cannot progress if concepts are not defined clearly. The current understanding of CE is ambiguous and multifaceted, and, therefore, conceptual and theoretical consolidation is highly welcome [11–13]. In response, this article has contributed to advancing the scholarly literature on CE through establishing some theoretical anchoring for the study of CBMs, providing some bases for the future academic development of CE thinking, which to date has been mostly influenced by non-academic stakeholders [102] and received little consideration by management scholars [94].

Conceptual and theoretical clarity is also relevant from a management practice perspective since a lack of it is among the barriers hindering CE implementation [103]. Furthermore, each of the theoretical perspectives used in this article has important implications for those organisations that are considering implementing CBMs. Particularly, they acknowledge the organisational dimension (resources, capabilities, and sustained competitive advantage) as well as the systemic (coevolutionary) and the within-system (ecological sphere) nature of BM innovation for circularity.

## Limitations

The repertoire of perspectives employed to advance the theoretical anchoring of CBMs, although spanning the organisational level and being system-oriented, is also limited. Nonetheless, it can be enriched with other lenses that in addition to those used here, can expand the theoretical anchoring of CBMs. Expanding the research horizon is particularly suited to the context of sustainability research. In fact, the complexity of sustainability challenges requires a multilevel research approach which includes micro, meso, and macro levels and several aspects of existing management theories (e.g. upper echelons theory, attention-based view, institutional theory) [104].

## Data Availability

Not applicable.
